# Change in Blink Rate in the Metaverse VR HMD and AR Glasses Environment

**DOI:** 10.3390/ijerph19148551

**Published:** 2022-07-13

**Authors:** Jungho Kim, Leehwan Hwang, Soonchul Kwon, Seunghyun Lee

**Affiliations:** 1 Immersive Content Device Technology Research Center, Kwangwoon University, Seoul 01897, Korea; jungh@kw.ac.kr; 2 Department of Plasma Bio-Display, Kwangwoon University, Seoul 01897, Korea; hlh0003@hanmail.net; 3 Graduate School of Smart Convergence, Kwangwoon University, Seoul 01897, Korea; ksc0226@kw.ac.kr; 4 Ingenium College, Kwangwoon University, Seoul 01897, Korea

**Keywords:** blink rate, metaverse, VR HMD, AR glasses, guideline

## Abstract

Blink rate, a major physiological response in humans, directly affects ocular diseases such as keratitis and dry eye. The blink rate in normal eyes appears at a constant frequency of 6–30 times per minute and is constant for each individual. In a previous study, the blink rate decreased when viewing content with high intensity and realism. Therefore, we tried to investigate the change in blink rate when viewing the content in VR HMD (virtual reality head-mounted display) and AR (augmented reality) glasses environments. We compared and analyzed the blink rate in four environments: natural state, viewing monitor, viewing VR HMD, and viewing AR glasses. Twenty-one participants (age, 26.87 ± 3.31 years) viewed the content for 1 min in four environments. One-way repeated ANOVA was used to analyze the blink rate changes. The study showed that the blink rate was decreased in the monitor, VR HMD, and AR glasses environments compared to that in the natural environment. Comparing the VR HMD environment with the AR glasses environment showed that the blink rate decreased in the VR HMD environment. The results of this study can be used for content use safety recommendations (guidelines for safe use of contents due to decreased blink rate) in the VR HMD and AR glasses environments, which are currently attracting attention in the metaverse.

## 1. Introduction

Humans receive information from the external environment through various sense organs. Among them, vision is a higher sense than the other senses and plays an important role in eliciting or aiding other senses. Most ways that humans acquire external information is transmitted through vision [1,2,3].

Eye blinking, a major human physiological reaction, is caused by the interaction between the levator palpebrae superioris muscle, which lifts the palpebral, and the orbicularis oculi muscle, which closes the eyelid. When the normally activated levator palpebrae superioris muscle is deactivated, and the orbicularis oculi muscle contracts, the eye closes. When the contraction of the orbicularis oculi muscle ceases and the levator palpebrae superioris muscle contracts, the mechanism that opens the eye results in eye blinking [4,5,6].

Blinking is an involuntary phenomenon that closes and opens the eyes at a speed of 100–400 ms [7]. In everyday life, blinking occurs unconsciously due to blinking suppression, which inhibits visual perception activity in the brain. The suppression time is approximately 200–250 ms, and it occurs from just before to immediately after blinking.

**Figure 1:**
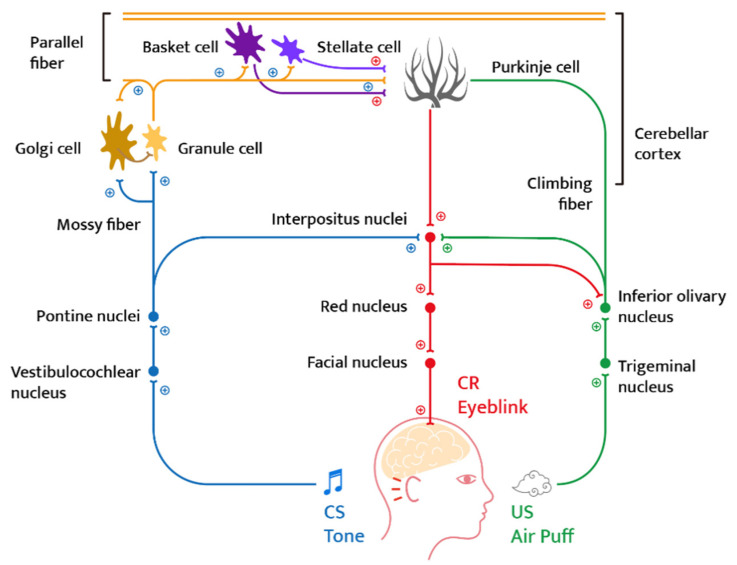
Essential neural circuitry of blink conditioning (reconstitution). (Source: Dominic T. Cheng et al. [8] “Eyeblink classical conditioning in alcoholism and fetal alcohol spectrum disorders”, 2015).

Blink types include spontaneous blink, reflex blink, and voluntary blink. A spontaneous blink is a natural eye movement that occurs without external stimuli and internal effort. This type of eye movement occurs in the prefrontal brainstem and occurs without conscious effort. A reflex blink occurs in response to an external stimulus. It occurs faster than a spontaneous blink and is generally caused by tactile stimulation, optical stimulation, or auditory stimulation. Blinking that occurs voluntarily is a means of communication or social interaction and can be maintained irregularly by controlling consciousness. Blinking can occur arbitrarily if the cornea needs lubrication or the eyes require protection in the case of fast visual movement and proximity. Voluntary blink has a larger amplitude than the reflex blink and uses the orbicularis oculi. Figure 1 shows the neural circuit that controls blinking (blue line: conditioned stimulus pathway, green line: unconditioned stimulus pathway, and red line: conditioned response pathway).

In individuals with normal vision, a spontaneous blink occurs at a certain frequency. The factors affecting the blink rate are eyelid condition, eye condition, disease status, presence of contact lenses, psychological state, surrounding environment, drugs, and other stimuli. The blink frequency ranges from 6 to 30 times/min [9,10].

The blink response directly affects ophthalmic diseases, such as keratitis and dry eye [11,12,13]. In addition to the ophthalmic factors, the tear film on the ocular surface is evenly dispersed during blinking to remove corneal irregularities and prevent corneal and conjunctival dehydration. Furthermore, the blink rate is decreased during the viewing of immersive content; therefore, it is necessary to study the relationship between the viewing of such realistic media and blink rate patterns.

In general, the blink rate tends to decrease during work compared with that at rest [14,15]. In addition, the blink rate tends to decrease during reading or demanding tasks, as these are more visually strict than the typical visual activities [16]. The higher the focal degree, the lower the blink rate [17,18]. According to various experiments related to blinking, the lead pilot has a lower blink rate with a shorter duration than that of the co-pilot in a simulated flight. Individuals driving on busy city roads have a lower blink rate than those driving on quiet, small city roads [19]. In the study by Kong et al., the blink rate was twice as high when looking at a white wall than when looking at a monitor or reading [20].

The brain focuses on acquiring information from the environment or image and tries to accept the information better by reducing the blink rate.

The recent method of display contents has the form of a metaverse, which is classified into virtual reality, augmented reality, lifelogging (mobile-based), and mirror world. As the metaverse is attracting attention as a means of replacing the internet, exponential market growth is expected in the future. With the development of immersive experience technology, the metaverse, a cyberspace, will establish itself as an expansion reality of the physical real world. In addition, as it is used as a digital world along with the real world, the usage time will gradually increase. Through virtual reality, one of the technologies to experience the metaverse, the distance between the display and the eyes has become closer. In addition, augmented reality services and lifelogging are accessed using mobile phones with smaller display sizes than existing TVs or monitors.

If the interest arousal due to the use of immersive contents based on the metaverse increases, it may manifest as a decrease in the blink rate, which may cause pathological effects on the eye.

In the study of Sheedy et al., ocular dryness is suggested as one of the external symptoms of stable fatigue [21], and xerophthalmia can be induced by a reduced blink rate. Ocular dryness is accompanied by visual disturbances, inflammation of the ocular surface, and ocular discomfort [22], thereby causing symptoms such as irritation, a burning sensation, and conjunctivitis.

This study proposes an environment to measure the blink rate. In addition, the effect of four environments (natural, monitor, virtual reality head-mounted display (VR HMD), and augmented reality (AR) glasses) on the blink pattern was assessed, and safety recommendations for the use of metaverse content were proposed accordingly.

### 1.1. Wearable Display of Metaverse (VR and AR)

The head-mounted display (HMD) is an interface device that allows human sensory organs such as the eyes and ears to experience a virtual environment. HMD is a device that comprises a high-resolution display, GPS, gyroscope, and wireless communication device. It recognizes the movements of the user and outputs a corresponding image [23,24,25].

The conception and development of the HMD concept began in 1968. See-through HMD, developed by Ivan E. Sutherland, is the world’s first instance of HMD use. The early HMD system required numerous pieces of equipment, such as a computer for graphic generation, a computer for trackers, and a computer for interaction. Therefore, it was not easy to develop HMD due to the low performance of the computer and the lack of software for development. The types of HMD, depending on the display technology, include an optical see-through display type, a video see-through display type, and a non-see-through display type.

#### 1.1.1. Optical See-through Display Type

**Figure 2:**
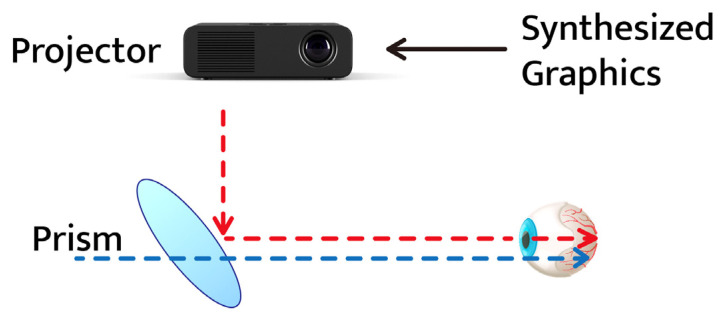
Optical see-through display type.

The optical see-through display type transmits real light through the HMD display [26,27]. This is a method in which the light passing through the display is combined with the image generated by the computer. In the early days, the transmitted light was reduced by half due to the use of a half-mirror. Recently developed models are shifting towards using projectors and prisms. The optical see-through type has the following advantage: the computer-generated image is output in a translucent state and does not block the user’s view. However, it is difficult to match the reality viewed by the user with the image generated by the computer. The optical see-through type display method is classified into a mobile phone mirroring method and an anchor method according to the augmentation technology. Figure 2 shows a schematic diagram of optical see-through display.

#### 1.1.2. Video See-through Display Type

**Figure 3:**
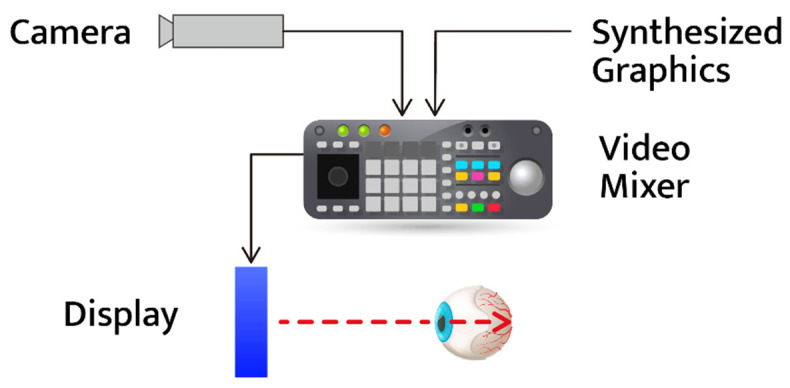
Video see-through display type.

The video see-through display type is a method of synthesizing an image acquired using a camera and an image generated by a computer and outputting it to the user [28]. It is possible to implement the video see-through type of display by attaching a camera to an HMD and a pair of glasses that are not manufactured as a video see-through type. However, there may be delays due to the operation of the camera to synthesize the images and graphics. Figure 3 shows a schematic diagram of video see-through display.

#### 1.1.3. Non-See-through Display Type

**Figure 4:**
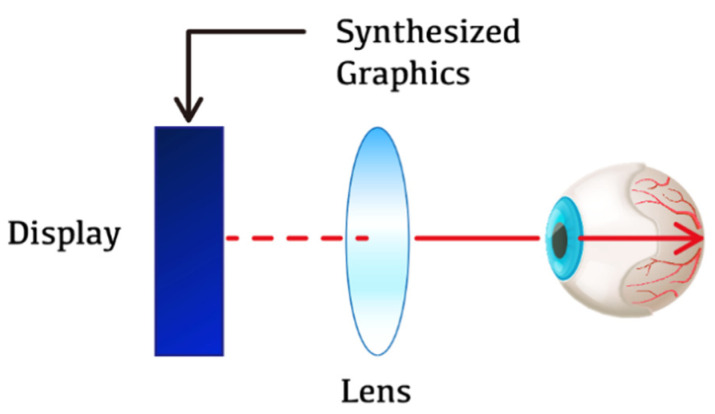
Non-see-through display type.

In the non-see-through display type, the view is blocked such that the outside environment cannot be visualized, and the user’s sense of immersion is high [29,30]. It uses an optical method that uses a lens to magnify the display at close range. This led to the popularization of HMD, which previously required expensive equipment. The position and posture of the user are tracked using an infrared sensor or an inertial measurement unit (IMU) sensor in the case of a non-see-through HMD. Figure 4 shows a schematic diagram of non-see-through display.

## 2. Materials and Methods

### 2.1. Participants

The participants selected in this experiment were 21 (male 16, female 5) individuals in their 20s and 30s (26.87 ± 3.31 years old) who provided informed consent to participate in the study. Participants had no ophthalmic, mental, or systemic diseases as well as far and near-corrected vision of 0.8 or higher, and no suppression on the worth 4 dot test.

Participants were recruited through an in-school notice for employees and students who were working and attending Kwangwoon University. Participants who completed the experiment were paid a designated participation fee.

### 2.2. Experimental Environment

Before starting the experiment, the participants completed physical condition and history questionnaires to check for factors that could affect the results. The items on the history questionnaire were divided into three categories: job, body use (exercise and gaming), and past ophthalmic history. Participants with factors affecting the experiment were excluded.

The experiments were performed in the natural, monitor, VR HMD, and AR glasses environments. After measuring the blink rate in the natural environment, the participants randomly performed an experiment in the four environments to prevent the order effect.

The blink rate was measured for a total of 2 min for stabilization and then for 1 min, except for 30 s before and after. For measurement accuracy, the participants were not informed about the measurement of the blink rate.

**Figure 5:**
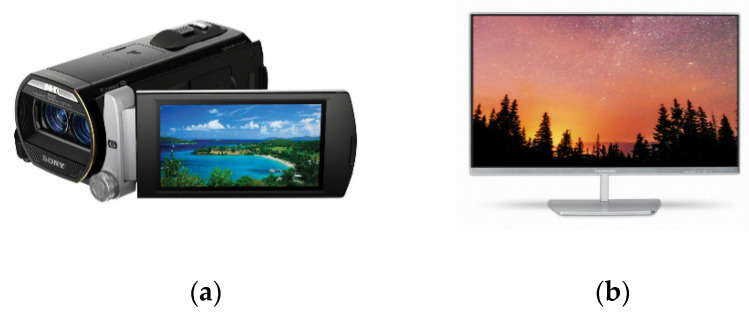

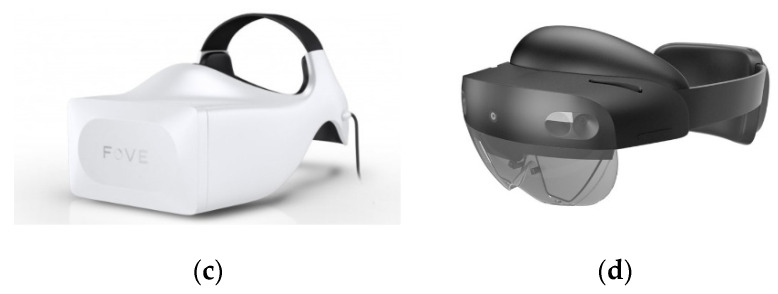
The equipment used in each environment to measure the blink rate: (**a**) TD20 camera; (**b**) Ultron 2457 monitor; (**c**) FOVE HMD; and (**d**) Hololens 2.

The blink rate in the natural state was measured using a camera (Model: TD20, Sony, Tokyo, Japan) without the examinee being made aware of the measurement. During the measurement, the examinee maintained a comfortable sitting position on the chair, and after the measurement was completed, the recorded file was checked to determine the blink rate. The measurement of blink rate in the monitor environment was performed using a 24 in monitor (Model: Ultron 2457, Hansung, Gimhae-si, Korea). The experiment was performed at a viewing distance of 1.0 m. It was recorded with the TD20 camera in the same way as the natural environment, and after the measurement was completed, the recorded file was checked to determine the blink rate. The content used ‘Grandmother’s Doll’. The blink rate in the VR HMD environment was measured using FOVE HMD to measure the blink rate while viewing content. The measurement position was adjusted using the Position Tracking Camera. It was measured by determining whether it was a blink using the ocular shown in the HMD eye tracking camera video debug tool. As for the content, ‘Grandmother’s Doll’ was used in the same way as the monitor environment. The blink rate in the AR glasses environment was measured using Hololens 2 while viewing the content. Measurements were performed in an environment in which 3D modeling content provided by Hololens 2 was freely operated. Figure 5 shows the equipment used in the experiment.

### 2.3. Research Data Analysis

Data analysis was performed using one-way repeated measures analysis of variances (ANOVA) on SPSS (Ver. 18.0 for Window, SPSS Inc., Chicago, IL, USA). Post hoc analysis (difference between groups) was performed using a paired-sample t-test. Statistical significance was set at *p* < 0.05 with a 95% confidence interval.

Repeated measures ANOVA is an extension of the paired-sample *t*-test, in which three or more measurements are acquired. It can be used when acquiring measurements from the same person at regular intervals and analyzing the results of repeated measurements depending on the time point.

## 3. Results

### 3.1. Comparison of the Blink Rate in the Natural Environment and Monitor Environment

**Figure 6:**
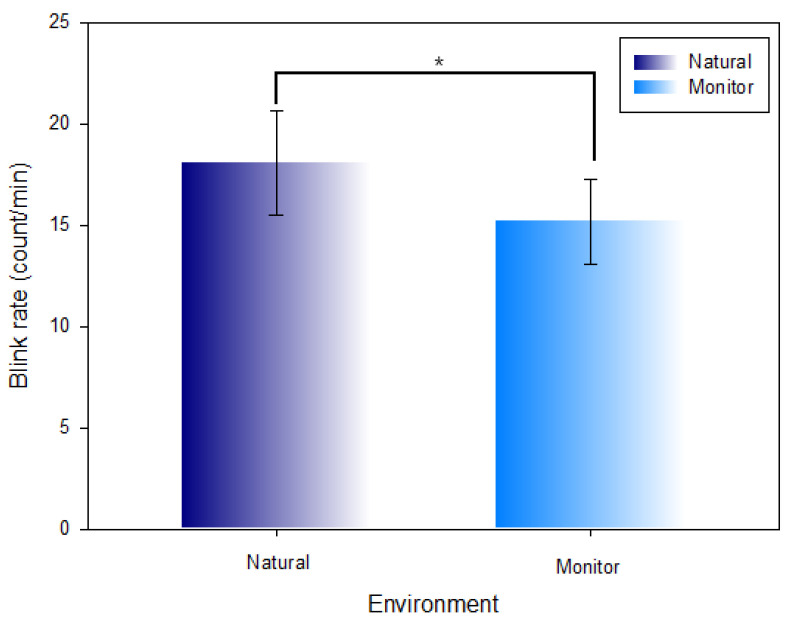
Comparison of the blink rate in the natural environment and monitor environment (*: *p* < 0.05).

Table 1 and Figure 6 show the comparison between the measured blink rate per minute in the natural environment (18.05 ± 2.56 count/min) and monitor environment (15.81 ± 2.09 count/min). The blink rate was comparatively decreased (Z = −4.050, $\chi^{2}$ = 57.466, *p* < 0.001) at a statistically significant level in the monitor environment.

### 3.2. Comparison of the Blink Rate in the Natural Environment and VR HMD Environment

**Figure 7:**
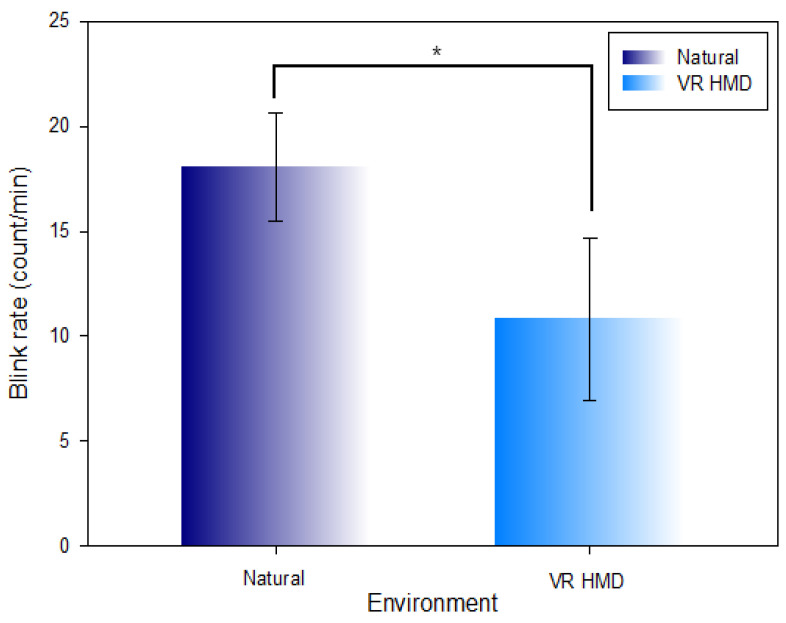
Comparison of the blink rate in the natural environment and VR HMD environment (*: *p* < 0.05).

Table 2 and Figure 7 show the comparison of the measured blink rate per minute in the natural environment (18.05 ± 2.56 count/min) and VR HMD environment (10.81 ± 3.89 count/min). The blink rate was comparatively decreased (Z = −4.017, $\chi^{2}$ = 57.466, *p* < 0.001) at a statistically significant level in the VR HMD environment.

### 3.3. Comparison of the Blink Rate in the Natural Environment and AR Glasses Environment

**Figure 8:**
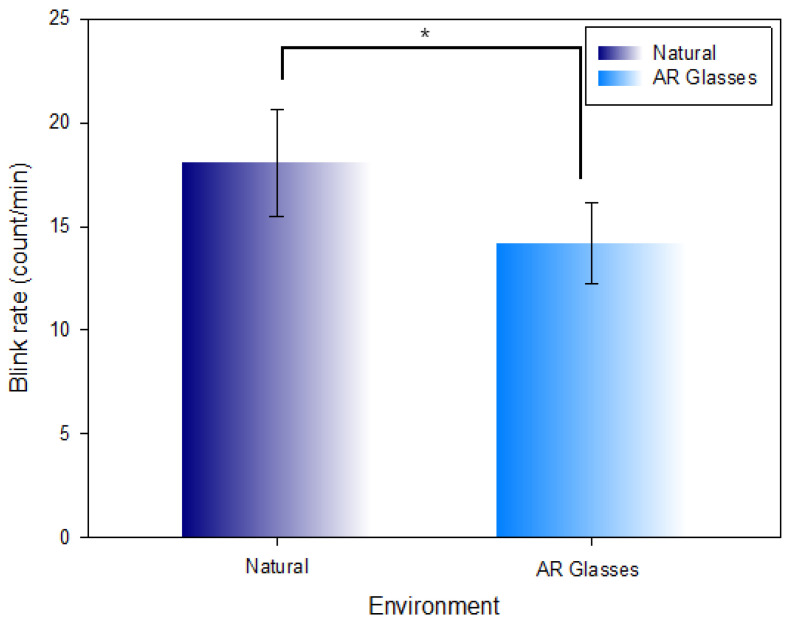
Comparison of the blink rate in the natural environment and AR glasses environment (*: *p* < 0.05).

Table 3 and Figure 8 show the comparison of the measured blink rate per minute in the natural environment (18.05 ± 2.56 count/min) and AR glasses environment (14.19 ± 1.97 count/min). The blink rate was comparatively decreased (Z = −4.047, $\chi^{2}$ = 57.466, *p* < 0.001) at a statistically significant level in the AR glasses environment.

### 3.4. Comparison of the Blink Rate in the Monitor Environment and VR HMD Environment

**Figure 9:**
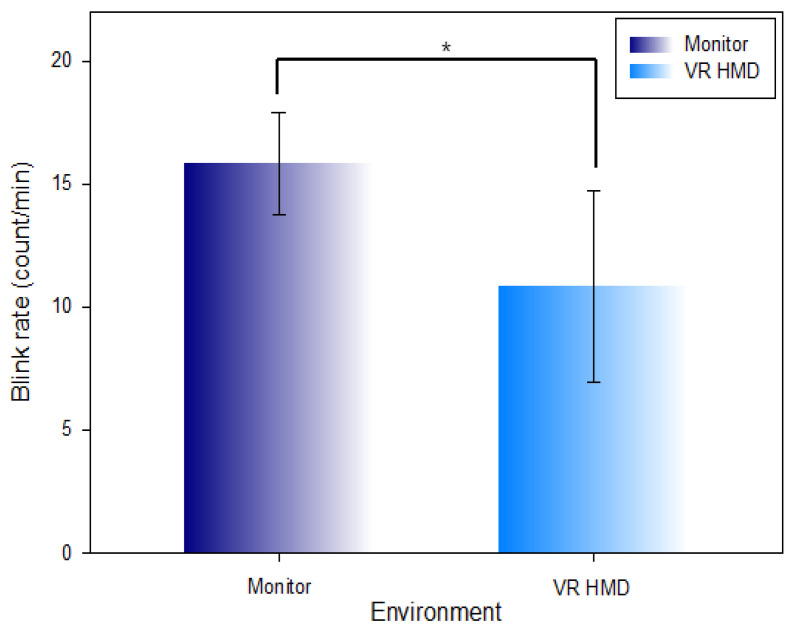
Comparison of the blink rate in the monitor environment and VR HMD environment (*: *p* < 0.05).

Table 4 and Figure 9 show the comparison of the measured blink rate per minute in the monitor environment (15.81 ± 2.09 count/min) and VR HMD environment (10.81 ± 3.89 count/min). The blink rate was comparatively decreased (Z = −3.834, $\chi^{2}$ = 57.466, *p* < 0.001) at a statistically significant level in the VR HMD environment.

### 3.5. Comparison of the Blink Rate in the Monitor Environment and AR Glasses Environment

**Figure 10:**
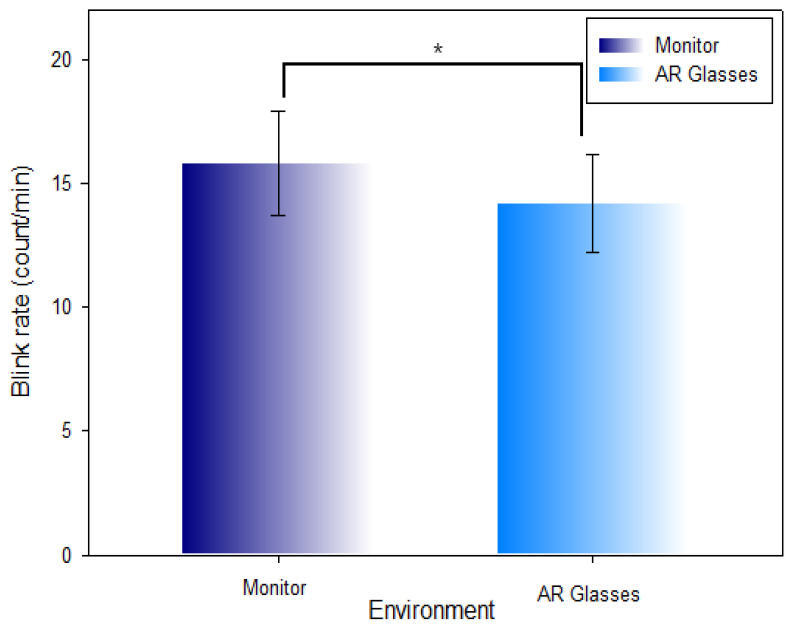
Comparison of the blink rate in the monitor environment and AR glasses environment (*: *p* < 0.05).

Table 5 and Figure 10 show the comparison of the measured blink rate per minute in the monitor environment (15.81 ± 2.09 count/min) and AR glasses environment (14.19 ± 1.97 count/min). The blink rate was comparatively decreased (Z = −4.102, $\chi^{2}$ = 57.466, *p* < 0.001) at a statistically significant level in the AR glasses environment.

### 3.6. Comparison of the Blink Rate in the VR HMD Environment and AR Glasses Environment

**Figure 11:**
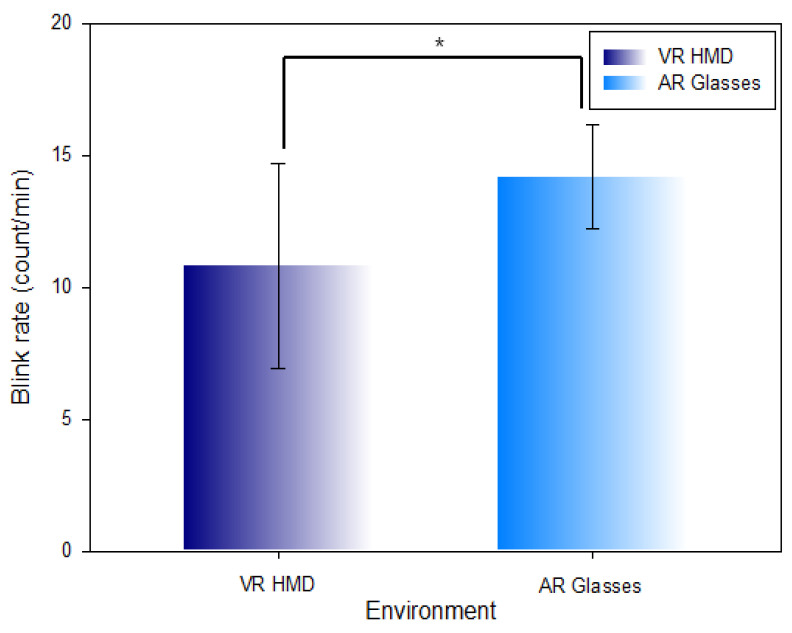
Comparison of the blink rate in the VR HMD environment and AR glasses environment (*: *p* < 0.05).

Table 6 and Figure 11 show the comparison of the measured blink rate per minute in the VR HMD environment (10.81 ± 3.89 count/min) and AR glasses environment (14.19 ± 1.97 count/min). The blink rate was comparatively decreased (Z = −3.148, $\chi^{2}$ = 57.466, *p* = 0.002) at a statistically significant level in the VR HMD environment.

## 4. Discussion

Blinking is an important factor in ocular physiology, and involuntary blinking is controlled by mechanisms associated with fatigue, loss of attention, and stress [31]. Blink speed and duration are used in various fields, such as assessing sleepiness [32], evaluating driver workload [33], diagnosing neurological disorders [34], and diagnosing Parkinson’s disease (reduced blink speed).

The blink rate decreases as the gaze intensity becomes stronger than the natural state. The decrease in the blink rate leads to the pathological action of the ocular, which requires great attention. Toda et al. [35] reported that 51.4% of patients complaining of stable fatigue experienced dry eyes, and 71.3% of patients with dry eyes experienced stable fatigue. The blink rate is reduced in environments that require near-field work and concentration [14,15]; therefore, the symptoms of dry eyes can worsen to a disease state when subjected to a recognition requirement task.

In this paper, compared with the natural environment, the blink rate decreased in the order of the monitor environment, the VR HMD environment, and the AR glasses environment. In addition, the comparison between the monitor environment, the VR HMD environment, and the AR glasses environment showed that the blink rate decreased in the order of the VR HMD environment, AR glasses environment, and monitor environment. It is judged that the metaverse environment increases the degree of interest and the gaze intensity. The same tendency was suggested in previous studies [13,14,15,16,17,18,19] that showed a decrease in the blink rate when performing visual activities with a higher intensity of gaze than natural visual activities.

In general, near-work, such as reading, causes visual fatigue, and the degree of fatigue varies depending on the environment. In this study, the blink rate was the smallest in a VR HMD environment where the physical viewing distance was close. In general, to increase the sense of immersion when experiencing immersive contents, the size of the display can be increased or the viewing distance can be set closer. As the sense of immersion increases, the concentration of contents increases, and the blink rate decreases. In addition, as the viewing distance approaches, the crystalline lens becomes contracted, and if this condition persists, visual fatigue occurs. The VR HMD-based contents environment completely blocks external reality stimulation so that only the contents displayed in HMD are visible. This characteristic is a factor that increases the sense of immersion in VR HMD contents. Although there is no burden of accommodation of the crystalline lens due to the optical convex lens embedded in the HMD, caution is required for long-term use because the physical distance between the ocular and the display is very close. It should also be noted that ocular diseases may occur as the time to use contents increases in a state in which the blink rate is reduced. The blink rate is affected, not visual fatigue or cognitive control [36]. Since the use of VR HMD for a long duration may result in the development of disease due to a decrease in the blink rate, it is necessary to refrain from using it for a long duration. If its use for a long duration is inevitable, taking a break is important. Compared with VR HMD, the monitor environment and AR glasses environment are relatively close to the natural environment and may be less burdensome to use for a long time. However, it is recommended to take 5–10 min of break for voluntary blinking when using them for 60–70 min.

## 5. Conclusions

With the metaverse becoming a hot topic, the rate of using equipment in the form of HMD and glasses to experience the virtual world and augmented reality has increased. This paper attempted to evaluate the change in the blink rate in the metaverse environment using experiments to measure the blink rate in the natural environment, monitor environment, VR HMD environment, and AR glasses environment. Considering the current status of research, the use of HMD contents with high immersion appears to be a factor that can decrease the blink rate. Blinking plays an important role in preventing dryness of the cornea, removing foreign substances through tears, and preventing blurring of the image due to eye movement. Therefore, users must be aware of the physiological role of blinking when using HMD.

In adults, it is recommended to stop using HMD for a while and give rest to the eyes if the individual experiences dryness. Children need sufficient guidance from their parents. Since individual blink rates are very important in ocular physiology, the blink rate during the use of special content should be observed as a major functional factor.

## Figures and Tables

**Table 1:** Comparison of the blink rate in the natural environment and monitor environment (*n* = 21).

	M ± SD	Z	$\chi^{2}$	*p*-Value
Natural	18.05 ± 2.56	−4.050	57.466	*p* < 0.001
Monitor	15.81 ± 2.09			

Unit: count/min, SD: standard deviation, Wilcoxon signed-rank test.

**Table 2:** Comparison of the blink rate in the natural environment and VR HMD environment (*n* = 21).

	M ± SD	Z	$\chi^{2}$	*p*-Value
Natural	18.05 ± 2.56	−4.017	57.466	*p* < 0.001
VR HMD	10.81 ± 3.89			

Unit: count/min, SD: standard deviation, Wilcoxon signed-rank test.

**Table 3:** Comparison of the blink rate in the natural environment and AR glasses environment (*n* = 21).

	M ± SD	Z	$\chi^{2}$	*p*-Value
Natural	18.05 ± 2.56	−4.047	57.466	*p* < 0.001
AR Glasses	14.19 ± 1.97			

Unit: count/min, SD: standard deviation, Wilcoxon signed-rank test.

**Table 4:** Comparison of the blink rate in the monitor environment and VR HMD environment (*n* = 21).

	M ± SD	Z	$\chi^{2}$	*p*-Value
Monitor	15.81 ± 2.09	−3.834	57.466	*p* < 0.001
VR HMD	10.81 ± 3.89			

Unit: count/min, SD: standard deviation, Wilcoxon signed-rank test.

**Table 5:** Comparison of the blink rate in the monitor environment and AR glasses environment (*n* = 21).

	M ± SD	Z	$\chi^{2}$	*p*-Value
Monitor	15.81 ± 2.09	−4.102	57.466	*p* < 0.001
AR Glasses	14.19 ± 1.97			

Unit: count/min, SD: standard deviation, Wilcoxon signed-rank test.

**Table 6:** Comparison of the blink rate in the VR HMD environment and AR glasses environment (*n* = 21).

	M ± SD	Z	$\chi^{2}$	*p*-Value
VR HMD	10.81 ± 3.89	−3.148	57.466	0.002
AR Glasses	14.19 ± 1.97			

Unit: count/min, SD: standard deviation, Wilcoxon signed-rank test.
